# Changes in Sports Participation across Transition to Retirement: Modification by Migration Background and Acculturation Status

**DOI:** 10.3390/ijerph14111356

**Published:** 2017-11-08

**Authors:** Johanna-Katharina Schönbach, Manuela Pfinder, Claudia Börnhorst, Hajo Zeeb, Tilman Brand

**Affiliations:** 1 Leibniz Institute for Prevention Research and Epidemiology–BIPS, 28359 Bremen, Germany; boern@leibniz-bips.de (C.B.); zeeb@leibniz-bips.de (H.Z.); brand@leibniz-bips.de (T.B.); 2 Health Sciences Bremen, University of Bremen, 28359 Bremen, Germany; 3 Department of General Practice and Health Services Research, Heidelberg University Hospital, 69120 Heidelberg, Germany; manuela.pfinder@med.uni-heidelberg.de; 4 Department of Health Promotion/Occupational Health Management, AOK Baden-Württemberg, 70191 Stuttgart, Germany

**Keywords:** retirement, life events, physical activity, sports, exercise, elderly, aging, migration, immigrants, acculturation

## Abstract

While total physical activity decreases over the life course, sports and leisure-time physical activity (LTPA) have shown to increase after transition to retirement. This paper aimed to investigate whether this change in sports participation differs (1) between non-migrant persons (NMP) versus persons with a migrant background (PMB), and (2) by acculturation status. Data was drawn from 16 waves of the German Socio-Economic Panel Study (SOEP) including 2664 NMP and 569 PMB. PMB were grouped according to acculturation status (integrated, assimilated, marginalised, separated), assessed regarding three dimensions (language, social interaction and identification). We applied multilevel logistic regression models, adjusting for sex, retirement age, socioeconomic status, health status and body mass index. Our results show that (1) transition to retirement led to an increase in the sports participation of NMP during the first 5 years and the subsequent 5 years after retirement. Changes in sports participation were modified by migration status: In PMB sports participation increased to a lesser extent than in NMP. (2) While sports participation of integrated PMB was not significantly different from NMP in the preretirement phase, sports participation among integrated PMB increased less after retirement compared with NMP. Marginalized and assimilated PMB did not show consistent sports participation patterns before retirement, but seemingly increased their sports participation less than NMP over the retirement transition. Separated PMB had particularly low levels of sports participation. Considering that LTPA is a key factor for healthy ageing, the increasing gap in levels of sports participation after transition to retirement indicates the need for interventions targeting physical activity of the older migrant population.

## 1. Introduction

The proportion of elderly in the population is rapidly growing. In 2050, 44% of the population in Europe is expected to be 60 years of age and older. This demographical shift is accompanied by an increase of age-related diseases as well as growing healthcare demands and economic costs [1]. Therefore, the modification of preventive health behaviours, such as physical activity (PA), becomes an increasingly important public health issue for healthy ageing [2,3,4,5,6,7,8].

PA decreases with age over the life course [9], but is susceptible to life events and transitions [10,11,12,13]. In fact, research examining PA across the transition to retirement [14,15,16,17,18,19,20,21,22,23,24,25,26,27] found an increase in sports and leisure-time physical activity (LTPA) in this stage of life [28]. Retirement—being accompanied by increased leisure time, changing social contacts and reorientation on health [29,30,31,32], might therefore present an opportunity to modify health behaviours and to target PA interventions [13].

The increase in LTPA after retirement was found to be an important key factor for an improved health status after retirement [33,34]. However, studies indicate that the effect is slightly higher in males than in females and greater among persons with a higher socioeconomic status (SES) [28]. At the same time, little is known on whether these changes in LTPA across the transition to retirement also exist among other population groups, such as immigrants [28]. Whereas changes in PA during retirement in non-migrants are affected by financial resources, social context and time availability [30,32], PA in migrants is additionally determined by cultural factors (religious beliefs, gender roles) and other factors related to acculturation, such as time since migration, generation and language [35,36,37]. Previous studies found that ethnic minority groups in Western countries are less physically active than the host population. For instance, migrants in Sweden, England and the Netherlands have shown lower levels of PA, even though there are indications that their PA possibly converges to the host population with increasing length of stay [38,39,40,41]. Similarly, results of a recent health survey in Germany indicated increased odds for physical inactivity among first-generation migrants, but not among second-generation migrants compared with the non-immigrant population [42].

Accordingly, previous research has stressed the importance of acculturation when examining PA in migrant populations, as greater acculturation was associated with an increased participation in exercise and PA [35,36,43]. Acculturation is the long-term process of adapting attitudes, values, customs, beliefs, behaviour and lifestyles that takes place as a result of contact between different cultural backgrounds [43,44]. This process affects at least three different dimensions of inclusion into society, i.e., shared practices (e.g., habits, language), social interaction (e.g., social networks), and identification (e.g., shared values, identities). Across the different dimensions, recent models assume that acculturation is a bilinear process of orientation towards culture of origin (CO) and orientation towards the host culture of the receiving country (HC). Based on these two orientations four acculturation categories are proposed: integration (CO orientation high, HC orientation high), assimilation (CO orientation low, HC orientation high), separation (CO orientation high, HC orientation low), and marginalisation (CO orientation low, HC orientation low) [44,45]. 

Henceforth, it can be expected that acculturation group status is also associated with different PA patterns. Migrants in the integrated and assimilated group are more likely to adapt their PA patterns towards the general population in the host country. Separated or marginalised migrants may be more influenced by the level of PA in the country of origin or may find fewer opportunities for sports participation that they find culturally appropriate.

To the best of our knowledge, this is the first study examining the effect of migration background and acculturation on changes in sports participation across retirement for the European context. We examined sports participation among German elderly persons transitioning from employment to retirement and explored possible interactions with migration background and acculturation status.

## 2. Materials and Methods

### 2.1. Data

For this longitudinal analysis, data were obtained from the German Socio-Economic Panel Study (SOEP) [46]. The panel started in 1984 using a household-based probability sample. It is the largest panel study and the largest regular survey of foreigners and immigrants in Germany. It is managed by the German Institute for Economic Research (DIW Berlin). All members of a survey household are interviewed (mostly face-to-face) on an annual basis, using pre-tested questionnaires. Topics include, among others, income, employment, education and health. Detailed information is given elsewhere [47]. The SOEP is approved as being in accordance with the standards of the Federal Republic of Germany for lawful data protection. In 2009, the German Council of Science and Humanities (Wissenschaftsrat) evaluated and approved the SOEP.

**Figure 1:**
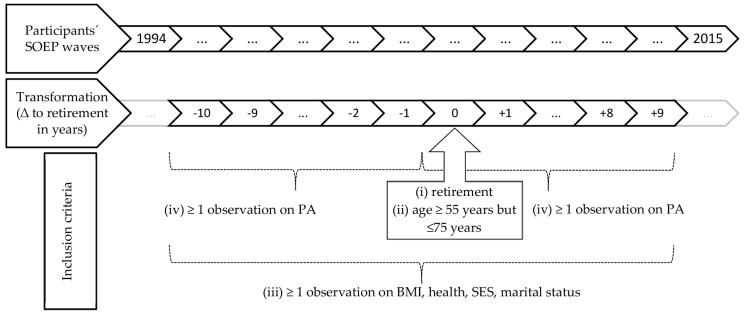
Sampling strategy with inclusion criteria (i) to (iv). Socio-Economic Panel Study (SOEP), physical activity (PA), socioeconomic status (SES), body mass index (BMI).

We used SOEP v32.1 [48], containing waves from 1984 to 2015 (Figure 1). From the original cohort, we identified persons that (i) were not retired when they entered the panel, but retired at some point at follow-up, and (ii) were ≥55 years but ≤75 years old at retirement entry. Around this individually varying point of retirement entry, we spanned the individual observation period that was limited to a maximum of 20 years, with 10 years both before and after retirement. Within this period, we considered all waves available for the single participants and restricted our sample to participants that (iii) provided at least one survey on all considered covariates (body mass index (BMI), health status, SES, marital status) within the 20-year observation period and (iv) provided at least one survey on PA within the 10-year observation period before and the 10-year observation period after retirement, respectively. This resulted in an overall sample of 3233 persons. Since sports participation was not assessed in all SOEP waves and participants attended in varying numbers of follow-ups, the numbers of waves participants contributed to the analysis varied from a minimum of two up to ten per study subject.

### 2.2. Variable Descriptions

#### 2.2.1. Sports Participation

Sports participation was assessed based on a single-item question. Participants were asked how often they participate in sports or exercise, based on four response options. The question does not cover work-related, housework-related or transportation-related PA. The variable was available in 16 waves. Participants were classified as being active if they stated to participate in sports at least “every week”, and they were classified as being inactive if they participate “every month”, “less often”, or “never”.

#### 2.2.2. Transition to Retirement

Transition to retirement used information on the self-reported previous year’s employment status. Participants were categorized as “not retired“ until they first stated to have retired during the previous year. From that point on (and including the previous year which was the actual year of retirement) they were categorized as retired. The variable assumes that, once retired, persons remain retired, thus not allowing for reversible retirement status.

We categorized transition to retirement into three phases: pre-retirement (year ten to year one before retirement), early retirement (zero to four years after retirement) and later retirement (five to nine years after retirement). We distinguished between early and later retirement to assess whether the change in sports participation was maintained over a longer period of time. 

#### 2.2.3. Migration Background

Information on participants’ country of birth, citizenship and their parents’ place of birth were used to determine the migration background of the participants [49]. To improve statistical power, we dichotomized the variable on migration background into “no migrant background” (NMP, *n* = 2664) or “migrant background” (PMB, *n* = 569), whereby the latter combines indirect migration background (*n* = 178, indicating that persons were born in Germany but have a migrant origin) and direct migration background (*n* = 391, indicating that persons immigrated themselves). Regions of origin of persons with a direct migration background were Turkey (*n* = 64), Ex-Yugoslavia (*n* = 50), Italy (*n* = 45), Greece (*n* = 35), Russia (*n* = 26), Poland (*n* = 23), Spain (*n* = 12), Romania (*n* = 12), and others (*n* = 95).

#### 2.2.4. Acculturation

Acculturation status was assessed across three dimensions of social inclusion as proposed by Esser [45]: Acculturation in terms of language, in terms of social interaction, and in terms of identification. For each of the dimensions, questions were included covering both CO and HC orientation. Language acculturation was assessed by two questions regarding German language proficiency and proficiency in the language of origin (talking and writing). Social interaction was covered by two questions asking whether the respondents were invited to the home of someone of German origin in the last 12 months (or the other way round) and whether respondents were invited or were visited by someone from their home country. In terms of identification, respondents were asked whether they felt like a German and whether they had a sense of belonging to their home country. In order to assess whether the three dimensions of acculturation differed in their association, the respondents were classified into acculturation groups for each dimension separately. The median split was used as a cut-off to categorize respondents as having a higher or lower orientation towards CO and HC for each dimension. The respondents were then classified into the four groups proposed by Berry [44]: integrated (CO orientation high, HC orientation high), assimilated(CO orientation low, HC orientation high), separated (CO orientation high, HC orientation low), and marginalised (CO orientation low, HC orientation low).

#### 2.2.5. Confounders

SES at retirement was generated based on the International Socio-Economic Index of Occupational Status (ISEI) as suggested by Ganzeboom [50]. The ISEI transforms information on the participant’s last occupational position into an index variable with values ranging from 16 to 90 with higher values indicating a higher SES. Marital status at retirement is based on participants’ self-reports. For the analysis, a dichotomous variable was created, categorizing participants into married or single, whereby the latter subsumed “single”, “widowed”, “divorced”, “separated” or “over 18 years and not with a partner”. Health status at retirement was self-rated by participants as being ”very good”, “good”, “satisfactory”, “poor” or “bad”. For this study, we merged the outer ones, resulting in a categorization of “poor”, “satisfactory” and “good”. BMI at retirement was calculated based on self-reported body height and weight (weight (kg)/height (m)^2^). Other socio-demographic variables were sex (male/female), as well as age at retirement. 

For all covariates, we used the participants’ information that was closest to their time of retirement, respectively. For all variables except BMI, this was the actual year of retirement in more than 95% of participants. BMI was derived from the actual year of retirement in 30.68% of the subjects, in 39.03% of the subjects from the following year, and in 30.29% of subjects from another year (translating into an average distance of 0.92 years after retirement).

### 2.3. Statistical Analysis

Characteristics of the study population are presented as mean values and standard deviations (SDs) for continuous variables and as proportions and frequencies for categorical variables. 

In the SOEP, repeated observations obtained from the same individual are correlated and also observations from individuals living in the same household cannot be assumed to be independent. Therefore, the effect of retirement on sports participation by migration background and acculturation status was analysed applying a multilevel logistic regression model with two levels to account for the clustered data structure, i.e., considering repeated observations being nested in participants who are in turn nested within households.

Model 1 examined the effect modification by migration status over the different phases of transition to retirement. It uses retirement, migration background and the interaction between both as main predictors, and adjusts for participants’ sex, SES, marital status, age at retirement, BMI and health status. Odds ratios (ORs) and 95% confidence intervals (CI), for being physically active during the two retirement phases versus before retirement, were calculated. By including an interaction term between retirement and migration background, we examined whether the odds of being physically active over the retirement phases varied by migration background. 

In addition, three models (Models 2, 3 and 4) examined the effect modification by acculturation status over retirement. Model 2 uses language acculturation, retirement, and its interaction term as main predictors; Model 3 uses social interaction acculturation, retirement, and its interaction term; Model 4 uses identification acculturation, retirement and its interaction term. All models were adjusted for participants’ sex, SES, marital status, age at retirement, BMI and health status. We examined whether the odds of being physically active over the retirement phases varied by acculturation status by including an interaction term between retirement and the respective acculturation variable. Due to the small sample sizes within the single acculturation groups and to ensure a stable model fit, here retirement was divided into two phases only: A pre-retirement phase (1 to 10 years before retirement), and a post-retirement phase (year 0 to year 9 after retirement). ORs and 95% CIs for being physically active after versus before retirement were calculated. 

Based on the results of the multilevel logistic regression, marginal means representing here the proportions of persons being physically active were estimated by migration background and acculturation status.

STATA (version 12, StataCorp, College Station, TX, USA) was used to analyse the data.

## 3. Results

### 3.1. Sample Characteristics

Overall, 20,597 observations of sports participation from 3233 subjects contributed to the analyses. NMP and PMB had similar characteristics with respect to sex, marital status, age at retirement and BMI (Table 1). However, more PMB rated their health status as being poor (32.34% vs. 24.10%) compared to NMP. Furthermore, SES scores differed substantially, being on average 38.62 (SD 16.23) in PMB and 46.63 (SD 16.89) in NMP. The distribution of acculturation groups differed across the three dimensions: In terms of language, the largest groups among PMB were classified as being integrated (30.96%) or marginalised (30.34%). In terms of social interaction, the largest groups were classified as being integrated (35.97%) or assimilated (34.16%). In terms of identification, most participants were either classified as being assimilated (39.19%) or separated (39.64%).

### 3.2. Sports Participation over Retirement Transition by Migration Status

Table 2 reports findings from the mixed-effects logistic regression (Model 1), comparing sports participation during the early and later phases of retirement with the pre-retirement phase, at the same time examining effect modification by migration status.

The results indicated that among NMP, transition to retirement led to an increase in sports participation during the early phase of retirement, which was maintained in the later phase of retirement. Meanwhile, sports participation among PMB increased to a lesser extent in both the early as well as later phase of retirement compared with NMP, as indicated by the interaction terms of retirement status and migration. The main effect for migration background in Model 1 suggests that PMB have decreased odds for sports participation compared with NMP even before retirement, but this association was not significant.

**Figure 2:**
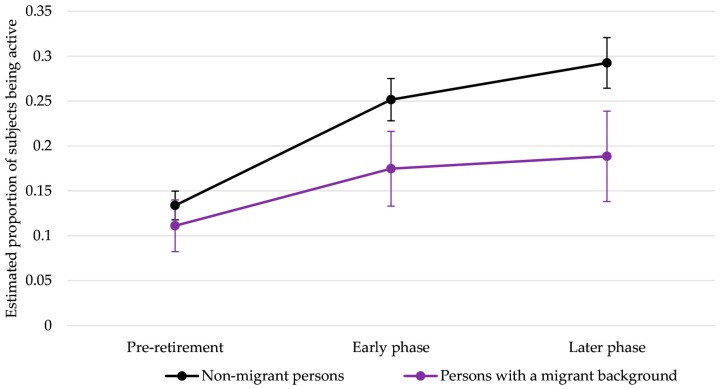
Estimated proportions of subjects participating in sports by retirement status adjusted for sex, age at retirement, SES, health status and BMI (marginal means with 95% CIs).

To ease interpretation of the interaction terms, Figure 2 displays the estimated proportions of NMP and PMB participating in sports in the pre-, early, and later phase of retirement after adjustment for age at retirement, sex, marital status, SES, health status and body mass index.

### 3.3. Sports Participation over Retirement Transition by Acculturation

Table 3 shows the results from the multilevel logistic regressions, comparing sports participation of the post- with the pre-retirement phase, at the same time examining effect modification by acculturation status.

Across all dimensions of acculturation, odds of sports participation in the pre-retirement phase did not significantly differ between integrated PMB and NMP. Concurrently, across retirement transition there was a smaller increase in sports participation among integrated PMB than among NMP, as indicated by the interaction terms of retirement status and acculturation (Model 2).

Sports participation was especially low in PMB classified as separated. In the language and in the identification dimension the main effect showed significantly lower odds for this group compared to NMP. In the social interaction dimension, the main effect for the separated group was not significant, but the significant interaction term indicated that the odds for sports participation increased to a lesser extent than in NMP (Model 3).

The results for PMB classified as assimilated and marginalised varied strongly across the three dimensions of acculturation. In the language dimension, the main effect for both groups indicated a lower level of sports participation compared to NMP. In the interaction dimension, sports participation levels of both assimilated and marginalised PMB were similar to NMP. In the identification dimension, levels of sports participation of both assimilated and marginalised PMB were higher than in NMP. When transitioning to retirement, a significantly smaller increase in sports participation was found for those marginalized in terms of identification (Model 4). Overall, there seemed to be a trend (though not being statistically significant) that sports participation increased less in assimilated and marginalised PMB than in NMP over the retirement transition.

**Figure 3:**
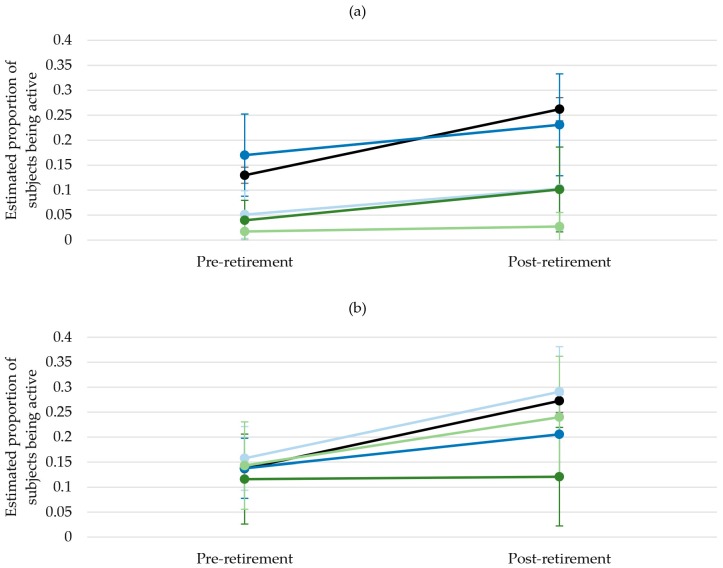

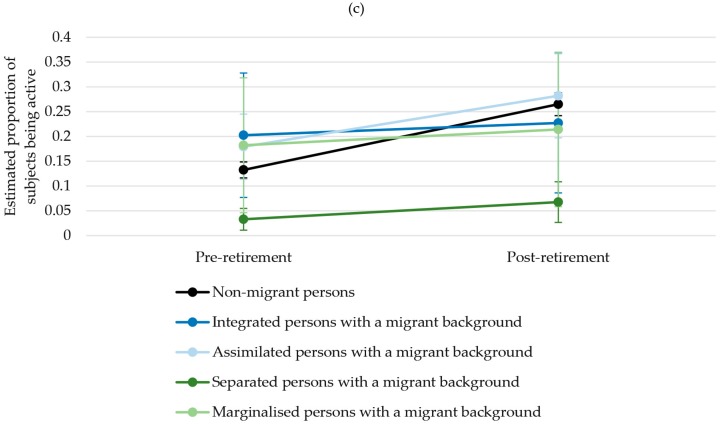
Estimated proportions of subjects participating in sports by retirement status adjusted for sex, age at retirement, SES, health status and BMI (marginal means with 95% CIs) for (**a**) Acculturation in terms of language; (**b**) Acculturation in terms of social interaction; (**c**) Acculturation in terms of identification.

Again, for a better interpretation the estimated proportions of participating in sports in the pre- and post-retirement phase among NMP and integrated, assimilated, separated and marginalised groups after adjustment for age at retirement, sex, marital status, SES, health status and BMI are illustrated in Figure 3.

## 4. Discussion

### 4.1. Main Findings

In this longitudinal study of elderly persons transitioning from employment to retirement, we found that sports participation increased during the first five years after retirement. This increase was maintained during the subsequent five years of retirement. The change in sports participation was modified by migration status, with PMB showing a smaller increase than NMP. We accounted for the heterogeneity of migrants in terms of acculturation status, which is a useful concept to distinguish between subgroups of PMB regarding their relationship to the host society. Whereas sports participation levels among integrated PMB were not significantly different from NMP in the preretirement phase, the increase in sports participation when transitioning to retirement was smaller among integrated PMB compared to NMP. For the marginalized and the assimilated groups, we did not find consistent sports participation patterns before retirement, but over the retirement transition there seemed to be a trend that sports participation increased less in assimilated and marginalised PMB compared to NMP. In the separated group, i.e., among persons with a strong orientation towards their culture of origin and a weak orientation towards the host culture, the odds of being active were particularly low.

### 4.2. Discussion of the Main Findings

It is evident that PA needs to be increased over the whole life course. Currently, four out of five adults are not sufficiently physically active in Germany [51]. Nevertheless, our observation of sports participation increasing after retirement indicates that retirement seems to be one of the life events where individuals might be especially receptive to PA promoting interventions [13]. Surprisingly, very few interventions target this time [52]. 

Our finding of sports participation increasing after retirement is consistent with the results of a previous systematic review [28]. These findings do not contradict the assumption that a systematic decrease occurs in total physical activity, as the increase in LTPA does not entirely compensate the loss of transport- and work-related activity [14].

As the first German study investigating the influence of migration background, our observations expand previous knowledge by adding that the increase in sports participation after retirement was smaller in PMB than in NMP. Considering the increasing share of PMB in the overall population in Germany and other high-income countries, it is important to assess their health behaviours in comparison to the host population to determine the need for tailored interventions to increase population health [53,54,55].

Nevertheless, generalizing our findings to other countries should be approached with caution. Studies from the U.S. context have reported inconsistent results, examining how PA over age and retirement transition varies between African Americans versus European Americans [17,26,28,56]. Differing migration patterns in terms of historical trends, migrant populations and immigration policies evoke difficulties of comparability not only between the U.S. and European context, but also between European countries [55,57,58].

Previous studies have identified possible barriers that limit PA participation in migrants, such as cultural and religious beliefs, environmental barriers, perception of health and socioeconomic challenges [35,36,37]. In our analysis, the gap between NMB and PMB persisted even after controlling for SES, indicating the importance to consider other factors related to migration background that function as determinants of physical activity. Similar observations have been made in previous research where the impact of migrant background on health status and health-related behaviour remained stable after adjusting for SES [42].

In migrant health research, it is important to note that the population with a migration background is not a homogenous group. We accounted for the heterogeneity of migrants in terms of acculturation status, applying Berry´s bilinear acculturation concept.

Previous research showed that pursuing assimilation was associated with most behavioural change, followed by integration being associated with selective adoptions of new behaviours, whereas separation was associated with fewest behavioural changes and marginalization with mainly undesirable behaviour changes [44]. Our own results indicate that even the integrated group had a smaller increase in sports participation over retirement than NMP. This could indicate that other underlying factors apart from acculturation play a role. For example, availability of sports and recreational facilities in the neighbourhoods may have an influence on sports participation. Again, heterogeneity needs to be considered, in that, acculturation leads to increased participation in some ethnic groups, but not in others [59]. Previous research has highlighted a life course perspective to understand migrant health and health behaviour, with the situation and social exposure of migrants before migration in their home country influencing their behaviour after migration in the host country [60]. However, studies on this issue usually fail to address the PA level of migrants in their home country [37].

It is unclear whether acculturation influences sports participation or vice versa [43]. Therefore, the suggested strategies are twofold. On the one hand, strategies that improve acculturation, such as language learning, might influence PA [43]. On the other hand, strategies that promote PA might influence acculturation, especially through migrant-sensitive and -inclusive rather than migrant-specific and -exclusive approaches [61]. Improving the access to sports clubs for the migrant population, by programs such as “Integration through sport” by the German Olympic Sports Confederation [62], could be a promising approach, particularly if elderly populations are consistently included.

### 4.3. Strengths and Limitations

A major strength of this study was the longitudinal panel design, in contrast to previous cross-sectional studies [19,21]. The SOEP is the largest panel study and the largest regular survey of foreigners and immigrants in Germany. It applies migrant-specific approaches, like translated questionnaires and specific migrant samples [63].

At the same time, our approach might hide cohort effects, as the group of 60-year-olds nowadays is more physically active than a group of 60-year-olds ten years ago [51].

The main limitation of this study concerns the variable on sports participation. Sports participation was self-reported and not objectively assessed, making the assessment imprecise and prone to recall bias. The need for more accurate measures of PA in large population studies in order to get more accurate estimates of associations has previously been emphasized [64]. Further, sports participation was only broadly categorized in SOEP (“every week”, “every month”, “less often”, or “never”). In reality, the amount and intensity of PA might vary considerably within each of these categories, and with its associated health impacts. For instance, the World Health Organization (WHO) recommends at least 150 min of moderate-intensity, or at least 75 min of vigorous-intensity aerobic PA per week [65]. Whereas the recommendation refers not only to activities during leisure time but also during house work, garden chores, transportation (e.g., biking or walking), or in any other context, we lacked information on other types of PA beyond sports.

A further potential measurement error affects the retirement variable. Misclassification might have occurred when persons did not retire fully but gradually over semi-retirement or went back to work after they retired. Nevertheless, our measurements are suitable for establishing general patterns.

An additional limitation is that we used covariates from waves that were closest to retirement although covariate values may have changed for some participants over the 20-year period. However, the covariates and sports questions were often assessed in alternating waves which was the reason for our approach. Our analyses on acculturation in PMB similarly have to be interpreted with caution. The number of persons within each acculturation group was quite small limiting the power to detect a moderating effect.

As sample size in this study did not allow for further stratification, we were not able to further differentiate PMB in order to account for the heterogeneity of migrants, for instance according to first- and second-generation migrants, length of stay, country of origin, sex and SES [55,57]. Future research should examine to what extent these factors affect the level of sports participation in PMB after retirement.

## 5. Conclusions

This study found that sports participation increased after retirement, whereby the increase was greater among NMP compared with PMB. Accounting for the heterogeneity of PMB in terms of acculturation status, we found that separated PMB had particularly low levels of sports participation. However, even the integrated PMB increased their sports participation over retirement to a lesser extent than NMP. Future research should examine the actually underlying reasons and mechanisms, as PA is a key factor for healthy ageing. Further, the increasing gap in levels of sports participation after transition to retirement indicates the need for interventions that improve access to sports clubs for the older migrant population and simultaneously support acculturational integration.

## Figures and Tables

**Table 1:** Characteristics of study sample.

Variable	Non-Migrant Persons (*n* = 2664)		Persons with a Migrant Background (*n* = 569)	
**Sex** (*n* (%))				
Male	1481	(55.59)	316	(55.54)
Female	1183	(44.41)	253	(44.46)
**Age at retirement** (mean (SD))	61.63	(2.85)	61.51	(2.95)
**Marital status** (*n* (%))				
Single	512	(19.22)	111	(19.51)
Married	2152	(80.78)	458	(80.49)
**SES** (mean (SD))	46.63	(16.89)	38.62	(16.24)
**Health status** (*n* (%))				
Poor	642	(24.10)	184	(32.34)
Satisfactory	1080	(40.54)	221	(38.84)
Good	942	(35.36)	164	(28.82)
**BMI** (mean (SD))	27.04	(4.24)	27.48	(3.99)
**Acculturation: Language dimension** (*n* (%))				
Non-migrant persons	2664	(100.00)	-	-
Integrated persons with a migrant background	-	-	100	(30.96)
Assimilated persons with a migrant background	-	-	62	(19.20)
Separated persons with a migrant background	-	-	63	(19.50)
Marginalised persons with a migrant background	-	-	98	(30.34)
**Acculturation: Social interaction dimension** (*n* (%))				
Non-migrant persons	2664	(100.00)	-	-
Integrated persons with a migrant background		-	159	(35.97)
Assimilated persons with a migrant background	-	-	151	(34.16))
Separated persons with a migrant background	-	-	64	(14.48)
Marginalised persons with a migrant background	-	-	68	(15.38)
**Acculturation: Identification dimension** (*n* (%))				
Non-migrant persons	2664	(100.00)	-	-
Integrated persons with a migrant background	-	-	50	(11.26)
Assimilated persons with a migrant background	-	-	174	(39.19)
Separated persons with a migrant background	-	-	176	(39.64)
Marginalised persons with a migrant background	-	-	44	(9.91)
**N (Observations)**	16,830		3767	

Data were missing for the language dimension of acculturation (*n* = 246), for the social interaction dimension of acculturation (*n* = 127), and for the identification dimension of acculturation (*n* = 125). Standard deviation (SD).

**Table 2:** Estimates from the mixed-effects logistic regression model on sports participation by migration background.

Variable	Model 1	
	OR (95% CI)	*p*-Value
**Retirement status** (ref: Pre-retirement)		
Early phase of retirement	2.66 (2.36–2.99)	<0.001
Later phase of retirement	3.49 (3.02–4.04)	<0.001
**Migration background** (ref: No migrant background)		
Migrant background	0.78 (0.55–1.11)	0.161
**Retirement status x Migration background**		
Early phase of retirement x Migrant background	0.71 (0.53–0.96)	0.027
Later phase of retirement x Migrant background	0.61 (0.42–0.89)	0.009
**N (observations)**	20,597	
***n* (persons)**	3233	
***n* (households)**	2515	

Pre-retirement: 1–10 years before retirement. Early phase of retirement: 0–4 years after retirement. Later phase of retirement: 5–9 years after retirement. Models were adjusted for sex, age at retirement, SES, health status and BMI. Odds ratio (OR), confidence intervals (CI).

**Table 3:** Estimates from the multilevel logistic regression models on sports participation by different dimensions of acculturation.

Variable	Model 2		Model 3		Model 4	
	Acculturation: Language Dimension		Acculturation: Social Interaction Dimension		Acculturation: Identification Dimension	
	OR (95% CI)	*p*-Value	OR (95% CI)	*p*-Value	OR (95% CI)	*p*-Value
**Retirement status** (ref: Pre-retirement)						
Post-retirement	2.92 (2.62–3.25)	<0.001	2.91 (2.62–3.24)	<0.001	2.92 (2.62–3.24)	<0.001
**Acculturation** (ref: Non-migrant persons)						
Integrated	1.47 (0.71–3.01)	0.298	1.01 (0.55–1.86)	0.981	1.86 (0.70–4.97)	0.213
Assimilated	0.31 (0.11–0.92)	0.034	1.22 (0.67–2.20)	0.515	1.55 (0.88–2.71)	0.130
Separated	0.24 (0.08–0.72)	0.011	0.80 (0.29–2.23)	0.668	0.19 (0.09–0.38)	<0.001
Marginalised	0.10 (0.03–0.29)	<0.001	1.06 (0.45–2.51)	0.891	1.58 (0.51–4.93)	0.427
**Retirement status x Acculturation**						
Post-retirement x Integrated	0.55 (0.33–0.93)	0.025	0.62 (0.39–1.00)	0.048	0.41 (0.20–0.85)	0.016
Post-retirement x Assimilated	0.80 (0.33–1.94)	0.627	0.92 (0.59–1.44)	0.725	0.72 (0.47–1.11)	0.138
Post-retirement x Separated	1.05 (0.45–2.44)	0.909	0.36 (0.16–0.83)	0.017	0.78 (0.43–1.43)	0.424
Post-retirement x Marginalised	0.56 (0.21–1.45)	0.230	0.76 (0.40–1.44)	0.395	0.44 (0.20–0.99)	0.047
**N (observations)**	19,076		19,625		19,715	
***n* (persons)**	2987		3106		3108	
***n* (households)**	2366		2426		2441	

Pre-retirement: 1–10 years before retirement. Post-retirement: 0–9 years after retirement. Models were adjusted for sex, age at retirement, SES, health status and BMI. Analytic sample sizes differ between models due to missing values on acculturation variables.
